# Sativex® (nabiximols) cannabinoid oromucosal spray in patients with resistant multiple sclerosis spasticity: the Belgian experience

**DOI:** 10.1186/s12883-021-02246-0

**Published:** 2021-06-22

**Authors:** Marie D’hooghe, Barbara Willekens, Valerie Delvaux, Miguel D’haeseleer, Daniel Guillaume, Guy Laureys, Guy Nagels, Patrick Vanderdonckt, Vincent Van Pesch, Veronica Popescu

**Affiliations:** 1 National MS Center, Vanheylenstraat 16, 1820 Melsbroek, Belgium; 2 Vrije Universiteit Brussel (VUB), Center for Neurosciences, Laarbeeklaan 103, 1090 Brussel, Belgium; 3 Department of Neurology, Antwerp University Hospital, Drie Eikenstraat 655, 2650 Edegem, Belgium; 4 University of Antwerp, Translational Neurosciences Research Group and Laboratory of Experimental Hematology, Vaccine & Infectious Disease Institute (VAXINFECTIO), Faculty of Medicine and Health Sciences, Universiteitsplein 1, 2610 Wilrijk, Belgium; 5 CHR de la Citadelle, Liège, Belgium; 6 CHU Liège – Centre Neurologique et de réadaptation fonctionelle (CNRF), Liège, Belgium; 7 University Hospital Ghent, Ghent, Belgium; 8 AZ Groeninge, Kortrijk, Belgium; 9 Cliniques Universitaires Saint-Luc, Brussels, Belgium; 10 University MS Centre, Noorderhart Hospital, Maesensveld 1, 3900 Pelt, Belgium; 11 University MS Center, U Hasselt, Noorderhart Hospital, Martelarenlaan 42, 3500 Hasselt, Belgium

**Keywords:** Multiple sclerosis spasticity, Observational study, Cannabinoid oromucosal spray, Treatment effect, Treatment satisfaction

## Abstract

**Background:**

This retrospective study evaluates patient-reported outcomes in patients with multiple sclerosis (MS) spasticity who were treated with a cannabinoid oromucosal spray (Sativex®, USAN name: nabiximols) after not sufficiently responding to previous anti-spasticity medications.

**Methods:**

Of 276 patients from eight centers in Belgium who began treatment prior to 31 December 2017, effectiveness assessment data were available for 238 patients during the test period of 4 to 8/12 weeks, and for smaller patient cohorts with continued treatment for 6/12 months.

**Results:**

Mean 0–10 spasticity Numerical Rating Scale (NRS) scores improved from 8.1 at baseline to 5.2 (week 4), 4.6 (week 8) and 4.1 (week 12). Mean EuroQoL Visual Analogue Scale (EQ VAS) scores increased from 39 at baseline to 52 (week 4), 57 (week 8) and 59 (week 12). Mean NRS and EQ VAS scores remained in the same 12 weeks’ range in patients with longer-term data. The average dose of cannabinoid oromucosal spray was 6 sprays/day. Most of the 93 out of 276 patients, with initial prescription (33.7%), who discontinued treatment by week 12 did so within the first 8 weeks, mainly due to lack of effectiveness. By week 12, 171 (74%) of the 230 effectiveness evaluable patients reported a clinically meaningful response, corresponding to ≥30% NRS improvement. The tolerability of cannabinoid oromucosal spray was consistent with its known safety profile.

**Conclusions:**

More than 60% of the patients with MS who started add-on treatment with cannabinoid oromucosal spray reported a clinically relevant symptomatic effect and continued treatment after 12 weeks.

**Supplementary Information:**

The online version contains supplementary material available at 10.1186/s12883-021-02246-0.

## Background

Spasticity, manifesting as chronic muscle rigidity usually worsened by spasms and cramps, is a frequent and often highly distressing symptom of multiple sclerosis (MS) [1]. Both the prevalence and severity of MS spasticity increase as the disease progresses, with about one-third of people with MS suffering moderate to severe spasticity after 10 years of disease despite conventional management [2]. In addition to stiffness and mobility restrictions [3], spasticity-associated symptoms such as pain, sleep disturbances, and bladder dysfunction contribute to a loss of independence and impair patients’ quality of life [1, 3–5].

The recommended treatment of MS spasticity is generally multi-modal, combining physiotherapy and pharmacotherapy [6]. Commonly used first-line pharmacological treatments for MS spasticity are baclofen, tizanidine and gabapentin [7]. However, patients’ ability to achieve effective doses of first-line oral medications may be limited by poor tolerability (e.g. undesirable effects on the central nervous system) [7], resulting in inadequate symptom relief in about one-third of patients [8]. Moreover, supporting evidence for the efficacy of most oral antispasticity agents is perceived to be scarce [9]. Assessments based on physician-rated instruments capture spasticity and spasticity-related symptoms as observed by the clinician at a certain moment in time, whereas a patient-rated 0–10 spasticity Numerical Rating Scale (NRS) offers a validated measurement of experienced symptom severity during the previous 24 h [10]. By anchoring the definitions of minimally clinically important difference (MCID; ≥ 20% improvement in baseline NRS score) and clinically important difference (CID; ≥ 30% improvement) to the patient’s global impression of change in spasticity severity, the 0–10 NRS provides a sensitive measure of treatment response in clinical trials and daily practice.

Based on randomized placebo-controlled trials demonstrating the efficacy and safety of the cannabinoid oromucosal spray mainly containing tetrahydrocannabinol (THC), cannabidiol (CBD), and other cannabinoid and non-cannabinoid components (Sativex®, USAN name nabiximols) for MS spasticity [11–13], the medicine was approved across the European Union (EU) and in other world regions as add-on therapy in patients with moderate to severe MS spasticity who have not responded adequately to first-line oral antispasticity medications [14]. Since then, several observational and registry studies have reported on the effectiveness and tolerability of the spray in daily practice [15–18], including data from 1615 Italian patients during the first 6 months after initial prescription of the cannabinoid oromucosal spray according to its approved label [18]. This large-scale analysis of the Italian Medicine Agency’s e-registry indicated that, under real-life conditions, about one-third of patients who begin treatment with this medication can expect to achieve a clinically meaningful and sustained improvement in MS spasticity at doses of about 6 to 7 sprays/day.

While temporarily approving the reimbursement of this cannabinoid oromucosal spray in 2016, Belgian health authorities requested data on NRS improvement after 4, 8 (or 12) weeks of use, average dosage and its evolution over time, frequency of and reasons for discontinuation, impact on quality of life, and tolerability. In adherence with these requests, the current analysis reports on the real-world effectiveness, safety and level of satisfaction with this cannabinoid oromucosal spray in Belgian patients with MS spasticity who were not sufficiently responding to conventional oral anti-spasticity medications.

## Methods

### Prescription, reimbursement and distribution

**Fig. 1:**
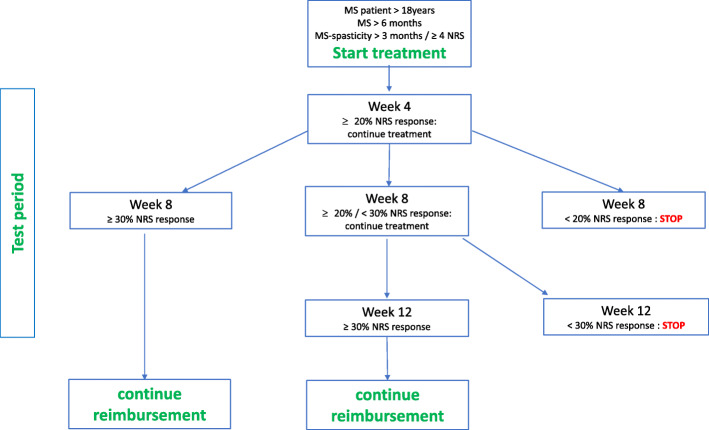
Reimbursement criteria for Sativex in Belgium

As this cannabinoid oromucosal spray is classified as a controlled substance in the European Union, its prescription and distribution must comply with Belgian narcotics legislation. Moreover, to obtain reimbursement, a prescription must fulfil additional requirements as outlined by the National Institute for Health, Diseases and Invalidity (NIHDI - www.riziv.fgov.be/www.inami.fgov.be):

- Reimbursement is only allowed for adult patients of at least 18 years, with a confirmed diagnosis of MS according to McDonald criteria [19] of at least 6 months’ duration who have been experiencing at least moderate spasticity (NRS score ≥ 4) for at least 3 months.
- Sativex® oromucosal spray should be prescribed as add-on treatment only when, at minimum, oral baclofen at an optimal dose and after an optimal treatment period has not been effective.
- Reimbursement is only approved when prescribed by neurologists with proven experience in the diagnosis and treatment of MS, as defined by the Belgian Ministry of Social Affairs [20].
- Patients prescribed Sativex® oromucosal spray must consent to sharing their data in a register and commit to maintaining a diary.
- Reimbursement is conditioned to a test period of a minimum 8 weeks and a maximum of 12 weeks during which a minimum clinical improvement in spasticity severity must be achieved, defined as an improvement from baseline of at least 20% (MCID) at week 4 and at least 30% (CID) at week 8 or week 12 on the 0–10 spasticity NRS. Only patients achieving a CID (≥ 30% NRS improvement) after week 8 or week 12 may continue treatment under reimbursement (Fig. 1).

### Data collection

This retrospective data collection involved data from patients who initiated treatment with cannabinoid oromucosal spray between 1 March 2016 and 31 December 2017 in eight MS centers in Belgium (Supplementary information). According to the Belgian legislation, there is no formal approval required by the Ethical committee when data are collected retrospectively and analysed anonymously. Data were collected from January 2018 to June 2018. All patients provided informed consent for their outcome data to be used for a Sativex® oromucosal spray register. Follow-up times varied according to the date of the baseline visit. Only patients with a baseline visit at least 4 weeks prior to data lock on 31 December 2017 were eligible for analysis.

Pseudonymized demographic data, including age and MS course, were collected retrospectively for each patient at baseline.

### Patient-reported outcomes

Patient-reported outcomes, collected at baseline, and after 4, 8, and/or 12 weeks of treatment (Fig. 1) and, if available, after 6, 9, 12 and 15 months of continued treatment, were included in the analysis. Outcomes of interest were overall treatment effect assessed with 0–10 spasticity NRS scores, medication usage pattern, treatment discontinuations, quality of life, and tolerability. Overall treatment effect was assessed by the change from baseline in 0–10 spasticity NRS scores. Medication usage pattern was assessed by the frequency distribution of sprays/day of this cannabinoid oromucosal spray based on patient diaries documenting use during the 7-day period prior to each clinic visit. The number and proportion of patients discontinuing treatment with this cannabinoid oromucosal spray, and reasons for discontinuation, were recorded. At each visit, patients rated their overall health-related quality of life on the visual analogue scale (VAS) component of the EuroQol five-dimensional (EQ-5D) instrument (EQ VAS), with scores ranging from zero (worst possible health) to 100 (best possible health). In line with the retrospective nature of the study and the effectiveness focus, tolerability was based on the collection of adverse events reported spontaneously by patients during treatment period.

### Responder definition

Onset of treatment effect was expressed by the number and proportion of patients showing a MCID (reduction of at least 20% in their spasticity 0–10 NRS score) after 4 weeks of treatment with the studied cannabinoid oromucosal spray (minimum threshold to continue treatment); and by the number and proportion of patients showing a CID at subsequent visits after 8 and 12 weeks of treatment (reduction of at least 30% in their spasticity 0–10 NRS score, minimum threshold to continue treatment). A 12-week assessment was mandatory only for patients with NRS improvement between 20 and 30% at week 8.

The responder rate at each visit was calculated as the proportion of patients eligible to start continued treatment versus the number of patients who started treatment before 1 November 2017, permitting assessment after the minimal test period of 8 weeks.

In selected patients with documented NRS data and visits for at least 8 weeks (minimal test period), in line with the Belgian reimbursement criteria, individual patient responses over the different time points were followed.

### Statistical methods

Total data collected per visit were used to calculate the mean and median of patient-reported 0–10 spasticity NRS scores, dosage, and EQ VAS score. At each designated time point and for each parameter, evaluable results were used as the reference for the descriptive analysis.

Treatment effects were assessed based on all available outcomes at each time-point.

With reference to specific Belgian reimbursement criteria, patient data were excluded for instances of deviations during the test period, e.g. visits diverting > 10 days from the mandatory 4, 8 or 12 weeks, missing NRS data, or continuous treatment for 12 weeks despite NRS improvement of < 20%.

## Results

A total of 276 patients with MS spasticity were prescribed add-on cannabinoid oromucosal spray between 1 March 2016 and 31 December 2017. Approximately three-quarters of patients were prescribed the medication in two of the eight participating centres.

As there was lack of follow-up data for 38 patients who initiated treatment in December 2017, NRS responses were available in 238 patients. Data were also collected for patients with 6 months (*n* = 180) and 12 months (*n* = 113) of continued treatment.

### Treatment effect

#### Patient-reported MS spasticity 0–10 NRS outcome (Table 1/Fig. 2a)

The mean patient-reported MS spasticity 0–10 NRS score improved from 8.1 (± 1.08) at baseline to 5.2 (± 1.85) after 4 weeks of treatment, to 4.6 (± 1.69) after 8 weeks of treatment and to 4.1 (± 1.78) after 12 weeks of treatment. NRS improvement was maintained in patients with available data at 6 months and 12 months, with mean scores of 4.3 (± 1.77) and 4.0 (± 1.92) respectively.

**Table 1:** Outcomes data by visit (the number of evaluable data per visit are used for calculations)

		Test treatment			Continued treatment	
	Baseline	Visit 1 (± 4 weeks)	Visit 2 (± 8 weeks)	Optional visit (± 12 weeks)	6 months	12 months
**N**	238	238	**230**^a^	^b^	180	113
**NRS**						
**eN**	238	229	188	*96*	103	60
Mean	8.1 ± 1.08	5.2 ± 1.85	4.6 ± 1.69	*4.1* ± *1.78*	4.3 ± 1.77	4.0 ± 1.92
Median	7.55 (2–10)	5 (0–10)	5 (0–9)	*4 (0–9)*	5 (0–9)	4 (0–9)
**VAS**						
**eN**	231	217	186	*93*	101	54
Mean	39 ± 22	52 ± 19	57 ± 20	*59* ± *20*	61 ± 18	64 ± 18
Median	40 (0–95)	50 (5–95)	60 (10–96)	*65 (10–93)*	65 (20–90)	70 (30–99)
**Number of sprays/day**						
**eN**		124	147	85	86	50
Mean		5.6 ± 2.31	5.9 ± 2.53	5.9 ± 2.39	6.0 ± 2.25	5.7 ± 2.08
Median		6 (1–12)	6 (1–12)	6 (1–12)	6 (1–12)	6 (1–12)

^a^According to Belgian reimbursement criteria (period 01 January 2016–28 February 2020) patients were assessed for response after a minimal 8 weeks of treatment

^b^12-week assessment was mandatory only for patients with > 20 and < 30% NRS improvement from week 4 to week 8, thus not possible to predict N

**Fig. 2:**
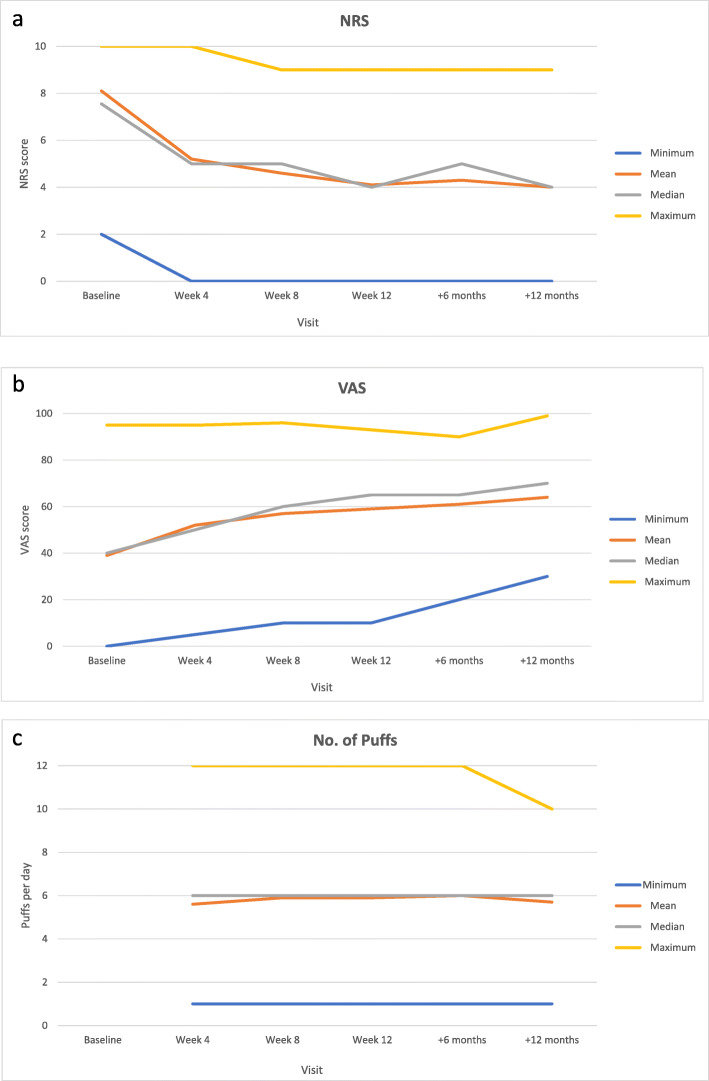
**a** NRS, **b** VAS and **c** number of sprays per visit (mean, median, minimum and maximum)

#### Health-related quality of life (Table 1/Fig. 2b)

The mean 0–100 EQ VAS score increased from 39 at baseline to 52 after 4 weeks of treatment with the study cannabinoid oromucosal spray and was 57 at week 8 and 59 at week 12. EQ VAS mean scores at 6 months (61) and 12 months (64) were stable in patients with available data.

#### Medication usage (Table 1/Fig. 2c)

The mean dose of the study cannabinoid oromucosal spray during the first 12 weeks after treatment start was approximately 6.0 sprays/day. The median daily dose during this test period was also 6 (range: 1 to 12) sprays/day. A mean and median dose of 6 sprays/day was maintained in the smaller cohort of patients with continued treatment for 6 or 12 months.

#### Treatment discontinuations

From the 276 patients that were prescribed add-on the studied cannabinoid oromucosal spray between 1 March 2016 and 31 December 2017, discontinuation during the first 12 weeks of treatment was documented for 93 patients (33.7%), mostly (*n* = 80) within 8 weeks of treatment start. The main reason for discontinuing treatment with THC:CBD oromucosal spray was perceived lack of effectiveness (*n* = 45). Eighteen patients discontinued treatment due to poor tolerability (not always the sole reason). Remaining patients (*n* = 30) discontinued treatment for other reasons and/or for reasons that were not documented.

#### Safety

A total of 39 spontaneously reported adverse events were recorded in the study source documents from 23 (9.7%) of 238 evaluable patients during the first 12 weeks after treatment start with cannabinoid oromucosal spray. The most frequent (unsolicited) adverse events reported were dizziness (8 events) and fatigue (6 events). Vomiting and bad taste were each reported by four patients 1 event/patient); and dry mouth and nausea were reported by two patients (1 event/patient) each. All other adverse events were each reported by one patient. Slightly more than half of all adverse events were reported during the first 4 weeks of treatment. No serious or unexpected adverse events were reported.

#### Response follow-up (Fig. 3)

Data from 8 of the 238 evaluable patients were excluded from the effectiveness analysis set because they had a baseline visit < 8 weeks before data lock point, resulting in NRS data for 230 patients. Data from 29 of these patients could not be used due to deviation with Belgian reimbursement criteria (deviation from scheduled visit time-points [*n* = 18], missing NRS data during the test period [*n* = 9] and continued treatment despite insufficient NRS improvement [*n* = 2]. While the follow-up data of these 29 patients have not been considered, they remained within the cohort for response analyses (calculation of percentages).

**Fig. 3:**
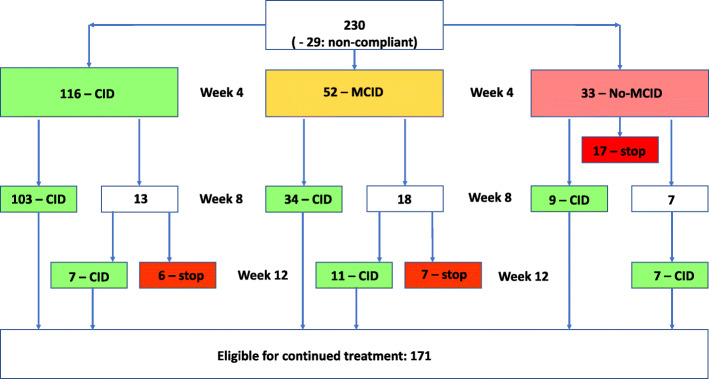
NRS response follow-up. Abbreviations according to Farrar et al. 2008. CID: Clinically important difference (> = 30% improvement on the NRS score) / MCID: minimal clinically important difference (> = 20% improvement on the NRS score) / no-MCID: < 20% improvement on the NRS score [21]. The 29 patients with deviating data collection have not been considered for response follow-up

After 4 weeks, 168/230 (73%) patients reported at least a MCID (≥ 20% NRS improvement) in spasticity severity, of whom 116 (50%) reported a CID response (≥ 30% NRS improvement). This improvement was maintained in 103/116 (89%) patients through week 8. Six of the 13 patients with a loss of initial response at week 8 discontinued treatment.

Of the 52/230 (22.6%) patients who reported a MCID but not a CID in spasticity severity at week 4, 34 reported a CID response at week 8. Eleven of the 18 patients without a CID at week 8 progressed to a CID response at week 12. The other seven of them stopped treatment due to loss of benefit.

At week 4, there were 33 (12.7%) non-responders of whom 17 patients stopped treatment. Nine patients reported a clinically meaningful NRS improvement (CID) at week 8 and the other seven reported a CID response only after 12 weeks of treatment.

Overall, 171 (74%) of 230 evaluable patients met the CID criteria for continued reimbursement of cannabinoid oromucosal spray according to Belgian criteria at week 12.

## Discussion

In this retrospective data analysis related to 276 patients with moderate to severe spasticity who were eligible to receive the studied cannabinoid oromucosal spray according to Belgian prescribing requirements, we obtained patient-reported outcomes in 238 patients. Treatment effectiveness was assessed using the 0–10 NRS, a validated patient-rated measure of patient-perceived spasticity severity [10], based on the Belgian criteria requiring 30% NRS improvement (CID) after minimum 8 or maximum 12 weeks of treatment to continue reimbursement. With a mean NRS score of 8.1 at baseline, the burden of spasticity was considerable in our patient cohort. Based on available outcomes at each time-point, the mean NRS score improved to 4.6 at week 8 (43% reduction), which appeared to be maintained in patients with available data at 6 months and 12 months.

The response analyses indicated that 168/230 evaluable patients (73%) achieved ≥20% NRS improvement after 4 weeks of treatment, including 116/230 (50.4%) patients reporting ≥30% NRS improvement. Our results align broadly with those of the Italian e-registry, where 62.5% of the baseline population reported ≥20% NRS improvement by week 4, including 25.1% with ≥30% NRS reduction [18]. Within 12 weeks after initial prescription, 171 (74%) of 230 evaluable patients in our cohort had ≥30% NRS improvement, thereby meeting the criteria for continuing treatment with cannabinoid oromucosal spray under Belgian reimbursement conditions.

The results of other patient-reported outcomes of interest in our study were consistent across participating centers and aligned with those of other studies. The double-blind randomized Sativex® as add-on therapy vs. further optimized first-line ANTispastics (SAVANT) study [22] reported a mean daily dose of 7.5 sprays/day at week 4 and 7.3 sprays/day after 12 weeks of treatment. Similar to the Italian prospective e-registry study which reported a mean daily dose of 6.8 sprays/day [18], we recorded an average dose of 6 sprays/day throughout treatment initiation and up to 12 months’ follow-up, suggesting no development of drug tolerance.

The treatment discontinuation rate in our data collection was similar to that reported in the Italian e-registry study (33.7 vs. 40%), as were timing (mainly within the first 8 weeks) and reasons for treatment discontinuation (mainly lack of effectiveness). It can be expected that the burden of treatment did not outweigh the benefit for some patients with sufficient NRS-response to continue treatment.

An interesting finding was patients’ self-rated health-related quality of life (HR-QoL) during treatment with the cannabinoid oromucosal spray. We are the first group to report these HR-QoL data during treatment in daily practice with such a long follow-up frame. While the mean 0–100 EQ VAS score of 39 at baseline suggests a substantial disease burden as perceived by patients, a 33% improvement was apparent by 4 weeks and maintained at 12 weeks. The 50% improvement in mean EQ VAS scores in a small cohort of patients treated for 6 and 12 months probably reflects the fact that only patients with real QoL benefits continued treatment. Similarly, the minimum EQ VAS score increased from 0 at baseline to 10 after 8–12 weeks of treatment, and to 20 and 30 after 6 months and 12 months, respectively. The ability of an intervention to improve HR-QoL in a chronic and progressive disease such as MS is remarkable. Even though the VAS component of the EQ-5D instrument may not be as comprehensive as the full questionnaire, it is a practical and useful tool to capture patients’ self-assessed health status in the time-constrained environment of everyday clinical practice [21].

The cannabinoid oromucosal spray was well tolerated in our retrospective cohort. Discontinuations due to adverse events were few, similar in nature to those described in the approved label, and similar to those reported in the Italian e-registry study. Therefore, most events should have been mild in intensity, occurred within the first few weeks of exposure and decreased in frequency with continued treatment beyond the first weeks. Tolerability to the cannabinoid oromucosal spray in this Belgian registry analysis was consistent with its known safety profile [14].

In our cohort, 16 patients (7.0%) with a clinically relevant response to nabiximols oromucosal spray at week 12 had not achieved 20% NRS improvement at week 4 of treatment. While the continuation of treatment with the studied cannabinoid oromucosal spray in these patients deviates from the Belgian prescribing requirements, it likely reflects clinical practice whereby treating neurologists apply their judgement to the management of individual patients. Spasticity patterns and variations in severity during the day may require adjustments of administration and dosing. From the authors’ perspective, some patients may require a more gradual up-titration of dosage for tolerance reasons resulting in a delayed onset of effect. Others who fail to achieve the numerical threshold of ≥20% NRS improvement by week 4 may respond with longer follow-up. In certain cases of patients with MS spasticity initiating treatment with cannabinoid oromucosal spray, an extended initial trial period beyond 4 weeks, with more frequent follow-up may facilitate fine-tuning of timing and dosing to achieve appropriate patient-tailored management. As of 1 March 2020, Belgian reimbursement authorities agreed to simplify the assessment schedule during the first period of reimbursement from the initial required assessment of NRS improvement at week 4, 8 (and if needed week 12), to assessment at week 4, followed by assessments as needed until week 16 in order to achieve the minimum 30% NRS improvement threshold required for continued reimbursement.

Study limitations need to be described. Compared with clinical trials conducted under controlled conditions, studies performed in daily clinical practice have limited ability to draw firm conclusions about safety and effectiveness. The retrospective data collection typically results in more missing data and potential biases when compared with prospective, randomized controlled trials or prospective observational studies. In our case, the availability of longer-term data was limited by the well-defined data collection period as per commitments to Belgian reimbursement authorities. As data after 12 weeks of treatment were not yet available at the time of collection lock point for patients who initiated treatment in late 2017, patient numbers with (retrospective) follow-up over an extended period were limited. Furthermore, it should be taken into consideration that many individuals with resistant MS–related spasticity experience impaired mobility, limiting the collection of complete data sets at designated time points, especially with regard to long-term data.

Nevertheless, we observed a similar response in Belgian patients when compared with other countries, suggesting differences in daily practices and healthcare systems do not impact effectiveness and tolerability of Sativex. Our data collection also included Health-related QoL assessments during treatment with Sativex, beyond 12 weeks.

## Conclusions

The alignment between this Belgian retrospective data analysis of patients receiving as add-on this cannabinoid oromucosal spray, the Italian prospective large e-registry study and other observational studies shows that the studied medication can be effective and safe in a meaningful proportion of patients with MS spasticity who do not respond sufficiently to other treatments in a real-world setting, While the 4-week trial period initially proposed in the context of clinical trials and then implemented in the approved label is useful to identify early non-responders, some flexibility is advised in the management of individual patients when improvements in spasticity-related symptoms fail to reach the 20% NRS improvement threshold within 4 weeks of start of treatment. Some patients may require tailored adjustments and assessment of early clinical response. In this regard, the extension in timelines for response assessment from 12 to 16 weeks in the new Belgian reimbursement criteria allows for a more patient-oriented responder assessment.

Data on total daily dose of medication used throughout our study is aligned with that of other, prospective observational studies suggesting that 6–7 sprays/day of this cannabinoid oromucosal spray provides effective relief of MS spasticity symptoms possibly extending over a period up to 12 months, without seeing an increase of the mean dose over time (no tolerance/habituation effect) and no cases of misuse/abuse were proactively reported. Our findings suggest that treatment responders may experience a sustained enhancement in health-related quality of life when they continue treatment.

In this retrospective cohort, more than 60% of the MS patients who started add-on treatment with the studied cannabinoid oromucosal spray (Sativex) reported improved spasticity and related symptoms, showed a clinically relevant symptomatic effect and went on with treatment after 12 weeks. Further investigation is recommended to determine predictive factors of treatment response.

## Supplementary Information

**Additional file 1.** Supplementary Information: Participating centers, neurologists and listed in order of their respective contributions in number of patients.

## Data Availability

The data that support the findings of this study are available from Almirall but restrictions apply to the availability of these data, which were used under license for the current study, and so are not publicly available. Data are however available from the authors upon reasonable request and with permission of Almirall.

## Abbreviations

CBD: Cannabidiol
CID: Clinically important difference
EQ VAS: EuroQoL Visual Analogue Scale
EQ-5D: EuroQol five-dimensional
EU: European Union
HR-QoL: Health-related quality of life
MCID: Minimally clinically important difference
MS: Multiple sclerosis
NRS: Numerical Rating Scale
THC: Tetrahydrocannabinol
VAS: Visual analogue scale
