# Supplementary material for: Sativex® (nabiximols) cannabinoid oromucosal spray in patients with resistant multiple sclerosis spasticity: the Belgian experience
Source: BMC Neurol. 2021 Jun 22;21:227. doi: 10.1186/s12883-021-02246-0 (PMC8218396; doi:10.1186/s12883-021-02246-0)
Supplement: Supplementary file 1 — Additional file 1. Supplementary Information: Participating centers, neurologists and listed in order of their respective contributions in number of patients. [file 12883_2021_2246_MOESM1_ESM.docx]

**SATIVEX^®^ (nabiximols) cannabinoid oromucosal spray in patients with resistant multiple sclerosis spasticity: the Belgian experience**

Marie D’hooghe^1*^, Barbara Willekens^2, 3^, Valerie Delvaux^4^, Miguel D’haeseleer^1^, Daniel Guillaume^5^, Guy Laureys^6^, Guy Nagels^1^, Patrick Vanderdonckt^7^, Vincent Van Pesch^8^ and Veronica Popescu,^9,10^

^1^National MS Center, Vanheylenstraat 16, 1820 Melsbroek, Belgium

^2^Antwerp University Hospital , Department of Neurology, Drie Eikenstraat 655, 2650 Edegem, Belgium

^3^University of Antwerp, Translational Neurosciences Research Group and Laboratory of Experimental Hematology, Vaccine & Infectious Disease Institute (VAXINFECTIO), Faculty of Medicine and Health Sciences, Universiteitsplein 1, 2610 Wilrijk, Belgium

^4^CHR de la Citadelle - Liège, Belgium

^5^CHU Liège – Centre Neurologique et de réadaptation fonctionelle (CNRF), Belgium

^6^ University Hospital Ghent, Belgium

^7^AZ Groeninge Kortrijk, Belgium

^8^Cliniques Universitaires Saint-Luc – Brussels, Belgium

^9^Universtity MS Centre, Noorderhart Hospital, Maesensveld 1, 3900 Pelt, Belgium

^10^University MS Center, U Hasselt, Noorderhart Hospital, Martelarenlaan 42, 3500 Hasselt, Belgium

* **Author for correspondence:** Marie D’hooghe

Electronic address: [marie.dhooghe@mscenter.be](mailto:marie.dhooghe@mscenter.be)

**Supplementary Information: Participating centers**, neurologists and listed in order of their respective contributions in number of patients.

| **Center** | **Responsible neurologists** | **Number of Patients** |
| --- | --- | --- |
| Antwerp University Hospital | Dr. Barbara Willekens | 8 |
| AZ Groeninge-Kortrijk | Dr. Patrick Vanderdonckt | 14 |
| Centre Neurologique et de réadaptation fonctionnelle (CNRF) − Liège | Dr. Daniel Guillaume | 15 |
| CHR de la Citadelle − Liège | Prof. Dr. Valerie Delvaux | 7 |
| Cliniques Universitaires Saint-Luc-Brussels | Prof. Dr. Vincent Van Pesch | 14 |
| National MS Center Melsbroek | Prof. Dr. Marie D’hooghe, Prof Dr Miguel D’Haeseleer, Prof Dr Guy Nagels | 103 |
| University MS Centre Pelt | Prof. Dr. Veronica Popescu | 113 |
| UZG Ghent | Prof. Dr. Guy Laureys | 2 |
